# Gastrointestinal Stromal Tumors (GISTs) Mimicking Primary Ovarian Tumors or Metastasizing to the Ovaries: A Systematic Literature Review

**DOI:** 10.3390/cancers16132305

**Published:** 2024-06-23

**Authors:** Gabriele Tonni, Andrea Palicelli, Maria Chiara Bassi, Federica Torricelli, Ilaria Vacca, Lorenzo Aguzzoli, Vincenzo Dario Mandato

**Affiliations:** 1 Department of Obstetrics and Neonatology, Istituto di Ricovero e Cura a Carattere Scientifico (IRCCS), Azienda USL-IRCCS di Reggio Emilia, Via Amendola 2, 42123 Reggio Emilia, Italy; 2 Pathology Unit, Azienda USL—IRCCS di Reggio Emilia, 42123 Reggio Emilia, Italy; andrea.palicelli@ausl.re.it; 3 Senior Librarian, Biblioteca Medica, Istituto di Ricovero e Cura a Carattere Scientifico (IRCCS), Azienda USL-IRCCS di Reggio Emilia, 42123 Reggio Emilia, Italy; bassi.mariachiara@ausl.re.it; 4 Translational Research Laboratory, Azienda USL-IRCCS di Reggio Emilia, 42123 Reggio Emilia, Italy; torricelli.federica@ausl.re.it; 5 Department of Obstetrics and Gynecologic Oncology, Azienda USL-IRCCS di Reggio Emilia, 42123 Reggio Emilia, Italy; ilaria.vacca@ausl.re.it (I.V.); lorenzo.aguzzoli@ausl.re.it (L.A.); mandato.vincenzodario@ausl.re.it (V.D.M.)

**Keywords:** gastro-intestinal stromal tumors, GIST, adnexal tumors, primary ovarian tumors, fallopian tube tumors, treatment, prognosis

## Abstract

**Simple Summary:**

This systematic review of the medical literature concerning the overlap between adnexal and gastrointestinal stromal tumors (GISTs) was designed to provide the overall number of previous observed cases and to calculate outcome prognosis, specifically the disease-free survival and overall survival. The impact of immunostaining and new chemotherapeutic medication such as Imatinib are also discussed.

**Abstract:**

**Background:** Gastrointestinal stromal tumors (GISTs) are a rare neoplasm, sometimes mimicking primary ovarian tumors (OTs) and/or metastasizing to the ovaries (M-OT). We performed a systematic literature review (SLR) of OTs and M-OTs, investigating differences in recurrence-free and overall survival. **Methods:** Our SLR was performed according to PRISMA guidelines, searching in Pubmed, Scopus, and Web of Science databases from inception until 21 April 2024. **Results:** Overall, 59 OTs (Group 1) and 21 M-OTs (Group 2) were retrieved. The absence of residual disease after surgery was achieved significantly in a higher percentage of patients with Group 1 GISTs (91.5%) compared with Group 2 GISTs (57.1%). Chemotherapy was more frequently administered to Group 2 patients (33% vs. 0%). Recurrence and deaths for disease were significantly more frequent in Group 2 than Group 1 cases (54.5% vs. 6.8%, and 37.5% vs. 9.8%, respectively). **Conclusions:** GISTs can rarely mimic primary ovarian cancers or even more rarely metastasize to the ovaries. Group 1 GISTs occurred in younger women, were not associated with elevated tumor markers, and had a better prognosis. In contrast, Group 2 GISTs occurred in older women, may exhibit elevated tumor markers, and presented a worse prognosis. However, no significant statistical difference for survival between the two studied groups was detected. Computed tomography scans can define the size of GISTs, which correlate to stage and prognostic risk classes. The gold standard treatment is complete surgical resection, which was achieved in almost all cases of Group 1 GISTs and in half of Group 2. Histopathology and immunohistochemistry are essential for the final diagnosis and guide chemotherapy treatment.

## 1. Introduction

Abdomino-pelvic masses are characterized by heterogeneous clinical presentations with clinical symptoms varying from pain (sometimes referred to the rectovaginal septum), dizziness, weight loss, or increased abdominal habitus due to either tumor volume or the association with ascites. Non-gynecological primaries metastatic to the female genital organs (including gastro-intestinal, pancreato-biliary or genitourinary neoplasms, mesothelioma, mesenchymal tumors, etc.) may be clinically misinterpreted as primary gynecologic tumors [1,2,3,4,5,6,7,8,9,10,11,12,13,14,15,16,17,18,19,20,21,22,23,24,25,26,27,28] and vice versa [29]. In particular, a few cases of gastrointestinal stromal tumors (GISTs) have been reported to mimic an ovarian tumor at presentation [1,2,4,5,6,8,9,10,11,12,13,14,16,18,19,20,21,22,23,24,25,26], while primary ovarian carcinomas metastatic to the gastro-intestinal tract have been rarely suspected to represent primary extraovarian GISTs [29].

GISTs are mesenchymal tumors, sometimes revealing aggressive behavior, thought to develop from the interstitial cells of Cajal bodies, which build a network in the gastrointestinal wall providing an interface between the autonomic nervous system and the muscle layer that they regulate like a pacemaker [11,29,30,31]. GISTs are uncommon tumors with a slightly higher incidence in Asia compared to Western countries (16–20 vs. 10–15 cases/year/million people). Epidemiology observations on prognostic variations of GISTs have been registered after the discovery of targeted chemotherapies (e.g., Imatinib) [32,33,34,35,36].

Most cases of GISTs are sporadic and comparable to familial ones both in terms of phenotype, histological profile, and molecular profile. About 5% of GISTs belong to genetic syndromes such as the Carney–Stratakis syndrome (dyad), Carney triad syndrome, family Neurofibromatosis type 1 (NF1), and primary familial GIST syndrome [35,36].

GISTs represent a very small portion of all gastrointestinal tumors (around 1%); they account for 80% of mesenchymal cancers and can develop from the stomach, small bowel, colon, and rectum but also from extra-gastrointestinal sites like the omentum, mesentery, retroperitoneum, and pancreas [12,37]. The most common presenting symptom is gastrointestinal bleeding, either acute (melena or hematochezia) or chronic (associated with anemia and its consequences), followed by abdominal pain, distension or discomfort, anorexia, or a palpable mass causing compression effects [38]. Up to a third of GISTs are diagnosed occasionally during surgery or imaging and, due to the delay in diagnosis, up to half of GISTs have already metastasized at the time of diagnosis [39]. The most common sites of metastasis are liver (65%) and peritoneum (21%); rarely, GISTs can metastasize to the bones, lungs, lymph nodes, skin, and, exceptionally, ovaries [40]. Ovarian metastases from GISTs can appear even many decades after resection of the primary tumor [41].

While GISTs have been traditionally classified according to World Health Organization (WHO) [42], this classification has changed over the past few years.

The current WHO classification of digestive system tumors (DST) supports the identification of risk categories for tumor progression based on mitotic index (<5 vs. ≥ 5 mitoses/5 mm^2^) and tumor size (cut offs: ≤2, >2 to ≤5, >5 to ≤10, >10 cm); prognosis seems influenced by the primary anatomic site of origin, as gastric GISTs may have a higher rate of local relapse than primary GISTs of the small intestine, while the latter are rarer but with a higher rate of abdominal dissemination and metastases [42]. This classification has strong clinical and prognostic implications as the two highest risk categories benefit from post-surgery adjuvant chemotherapy with tyrosine kinase inhibitors such as Imatinib or other agents in case of non-responder patients [43]. Tumor stage (AJCC/TNM) also has a prognostic impact, including tumor size (same cut offs for WHO risk categories), lymph nodes (rare), and/or distant metastases; moreover, two different staging systems are used for gastric/omental GISTs vs. GISTs of other primary sites [42,44]. Usually, intestinal and succinate dehydrogenase (SDH)-deficient GISTs are more unpredictable [42]. Tumor rupture is an additional relevant prognostic factor [42]. Immunohistochemistry (c-KIT, DOG-1) is relevant for the histologic diagnosis and molecular analysis (especially of *KIT* and *PDGFRA* genes) and can allow prognostic risk stratification and influence Imatinib dose modulation [42].

We have performed the first systematic literature review of GISTs mimicking primary ovarian tumors (GIST-OTs, Group 1) and/or metastasizing to the ovaries (GIST M-OTs, Group 2) at presentation. We analyzed clinical and histopathological prognostic factors to identify any differences between Groups 1 and 2, discussing the main differential diagnoses.

## 2. Methods

### 2.1. Systematic Review of the Literature

**Figure 1:**
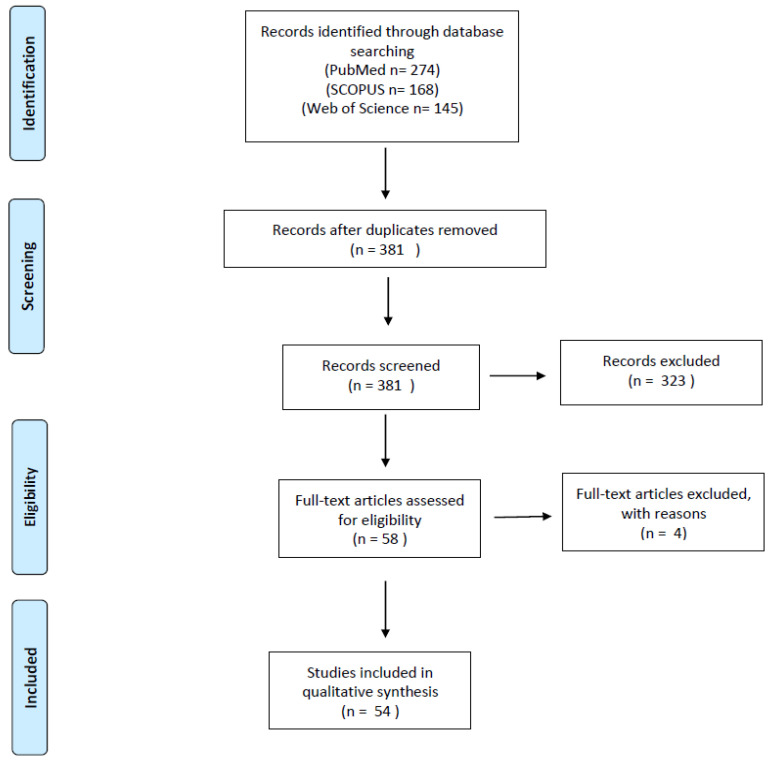
PRISMA flow-chart of our systematic literature review.

The electronic search was performed in agreement with the PRISMA (Preferred Reporting Items for Systematic Reviews and Meta-Analyses) guidelines for systematic literature reviews (http://www.prisma-statement.org/; accessed on 21 April 2024) (Figure 1). This systematic review of the medical literature has not been registered.

Our retrospective observational study was conducted via the PICO process:

- Population: human patients with GISTs mimicking a gynecological primary or metastasizing to at least 1 ovary at presentation;
- Intervention: any;
- Comparison: none;
- Outcomes: clinical outcomes (status at last follow-up, and survival and recurrence rates).

We searched for ((GIST OR GISTs OR “gastro-intestinal stromal tumor” OR “gastro-intestinal stromal tumour” OR “gastro-intestinal stromal tumors” OR “gastro-intestinal stromal tumours”) AND (ovary OR ovarian)) in the PubMed (all fields; 274 results; https://pubmed.ncbi.nlm.nih.gov, accessed on 21 April 2024), Scopus (Title/Abstract/Keywords; 168 results; https://www.scopus.com/home.uri, accessed on 21 April 2024), and Web of Science (all fields; 145 results; https://login.webofknowledge.com, accessed on 21 April 2024) databases. No limitations or additional filters were set. The bibliographic research ended on 21 April 2024.

We applied the following criteria:

- Eligibility/inclusion criteria: studies describing cases of patients with GISTs presenting as a primary ovarian tumor or metastasizing to one or both ovaries.
- Exclusion criteria: unclear diagnosis; ovarian metastases not from GISTs; results not analyzable (data too aggregated).

Two independent authors removed the duplicates and checked the titles and abstracts of all the retrieved results (*n* = 381). By applying the eligibility/inclusion and exclusion criteria, they selected 58 relevant eligible papers [1,2,4,5,6,7,8,9,10,11,12,13,14,16,18,19,20,21,22,23,24,26,30,41,45,46,47,48,49,50,51,52,53,54,55,56,57,58,59,60,61,62,63,64,65,66,67,68,69,70,71,72,73,74,75,76,77,78]. The data of 5 articles were retrievable only by abstracts [21,45,46,47,48], while the remaining papers were retrieved in full-text format, and their reference lists were screened for additional relevant studies. Four papers were excluded from the main analysis as they reported too aggregated or too scant/unclear data [46,48,49,50]. The remaining 54 papers were finally included in our study [1,2,4,5,6,7,8,9,10,11,12,13,14,16,18,19,20,21,22,23,24,26,30,41,45,47,51,52,53,54,55,56,57,58,59,60,61,62,63,64,65,66,67,68,69,70,71,72,73,74,75,76,77,78]. The extracted results were checked and confirmed by two other authors.

### 2.2. Statistical Analysis

Data collection was study- and case-related. Age of the patient, clinical symptoms, laboratory and diagnostic investigations such as ultrasonography (US), computed tomography (CT) and magnetic resonance imaging (MRI), immunohistochemistry, histopathology, surgical treatment, chemotherapy, and follow-up have been considered for clinical presentation and calculation of outcome. Categorical variables were analyzed as frequencies and percentages, whereas continuous variables were analyzed by ranges and mean values.

## 3. Results

Statistical analysis was performed using R Foundation for Statistical Computing (R-4.1.3, Vienna, Austria). Associations between clinical and pathological parameters were assessed by Fisher’s exact test for categorical variables and by Kruskal–Wallis test for continuous variables. Non-parametric distribution of all the continuous variables was previously evaluated by Shapiro test. Associations were considered statistically significant with a *p*-value lower than 0.05.

Overall, 59 cases of Group 1 GISTs (Table 1 and Table 2) [1,2,4,5,6,7,8,9,10,11,12,14,16,18,20,21,22,23,24,26,47,51,54,57,58,59,61,62,63,64,66,68,70,71,73,74,75,77] and 21 cases of Group 2 GISTs were retrieved (Table 3 and Table 4) [13,19,41,45,52,54,55,59,60,64,68,71,75,76]. The statistical analysis of the clinical variables of both studied groups is shown in Table 5.

### 3.1. Age

Globally, GIST patients had a mean age of 55.8 years (standard deviation; SD: ±14.6 years). The mean age of Group 1 patients (56.9 years; SD: ±14.8 years) was slightly higher than Group 2 women (52.1 years; SD: ±13.7 SD years). Data were missing in two Group 1 and four Group 2 cases.

### 3.2. Symptoms

Symptoms due to tumor mass such as abdominal distension, pain, mass, or discomfort, followed by constipation, lower extremity edema, or deep vein thrombosis, were present in 89% of Group 1 and in 70% of Group 2 patients. Gastrointestinal symptoms such as intestinal changes, stomach pain, nausea, bloating, anorexia, weight loss, and excessive gas were present in 21% of Group 1 and in 15% of Group 2 patients. Symptoms due to physical decay such as general malaise, anemia, weakness in lower limbs, fatigue, shortness of breath, and dehydration were present in 20% of Group 1 and in 10% of Group 2 patients. Ascites were reported in 10% of Group 1 and 15% of Group 2 patients.

### 3.3. Preoperative Imaging

Information about preoperative imaging was reported for 60/81 (70.1%) patients. CT was performed in 51/60 (85%) patients, being associated with MRI or US in 2/51 (3.9%) and 3/51 (5.9%) cases, respectively. MRI was performed alone in 7/60 (11.7%) patients, associated with US in 1/7 (14.3%) cases. Only 1/60 (1.7%) patients underwent exclusively US.

### 3.4. Tumor Markers

Ca-125 was increased in 21 (40.4%) Group 1 and 5 (71.4%) Group 2 patients (total: 26 cases, 44.1%). Conversely, Ca-125 was normal in 31 (59.6%) Group 1 and 2 (28.6%) Group 2 patients (total: 33 cases, 55.9%). When tested, CEA was normal in all the Group 1 (*n* = 18) and Group 2 (*n* = 5) patients. Finally, Ca-19.9 was increased in one patient for each Group (Group 1: 1/14, 7.1%; Group 2: 1/2, 50%; total: 2 cases, 12.5%) (Table 5).

### 3.5. Surgery

All 57 (100%) Group 1 and 18 (100%) Group 2 patients underwent surgery. Data were missing in two Group 1 and three Group 2 cases. Overall, there was no residual disease after surgery in 51 (83.6%) GIST cases; this result was achieved more frequently in Group 1 (43 cases, 91.5%) than Group 2 patients (8 cases, 57.1%). Instead, there was residual disease after surgery in four (8.5%) Group 1 and six (42.9%) Group 2 patients (overall, 10 cases, 16.4%). Data were missing in 12 Group 1 and 7 Group 2 cases (Table 5).

### 3.6. Other Treatments

Chemotherapy was administered to 3/9 (33.3%) Group 2 patients and no (0%) Group 1 patients (overall, 3 cases, 5.7%). When data were available, radiotherapy was not performed in any patients of the two groups (Group 1: 33 cases; Group 2: 5 cases). Overall, Imatinib was administered to 39/58 (67.2%) patients, including 8/9 (88.9%) Group 2 patients and 31/49 (63.3%) Group 1 cases (Table 5).

### 3.7. GIST Characteristics

The mean size of the tumor was 15.9 cm (SD +/- 7.6 cm) in Group 1 and 12.1cm (SD ± 6.2 cm) in Group 2 cases. Overall, the mean size was 15 cm (SD ± 7.4 cm) in GISTs. Data were missing in two Group 1 and three Group 2 GISTs.

Overall, the most frequent site of origin was the small intestine (59 cases, 78.7%), followed by the colon (10 cases, 13.3%) and stomach–omentum (6 cases, 8%). In particular, the small bowel was almost equally involved in both groups (45 Group 1, 78.9% vs. 14 Group 2, 77.8%), while the colon was slightly more frequently involved in Group 1 (9 cases, 15.8% vs. 1 case, 5.6%) and the stomach–omentum site was more frequently affected by Group 2 in terms of percentages but equal in absolute number of cases (3, 16.7% vs. 3, 5.3%). Data were missing in two Group 1 and three Group 2 GISTs (Table 5).

### 3.8. Histological Findings

Most GISTs were of spindle cell type (*n* = 36, 66.7%), while only 4 (7.4%) were epithelioid GISTs and 14 (25.9%) were mixed spindle and epithelioid GISTs. Data were missing in 24 GISTs overall. Unusual features included dedifferentiated areas (Group 1, case 3) [12], pleomorphic/severely atypical cells (Group 1, cases 4 and 49) [6,7], signet-ring cells (Group 2, case 72) [41], and myxoid features (Group 1, cases 51 and 55) [2,11]. No statistical significant differences were seen (*p* = 0.270, NS) (Table 5).

Necrosis was observed in 18 (85.7%) Group 1 and in 10 (83.3%) Group 2 GISTs (total: 28 cases, 84.8%), being absent in 5 (15.2%) GISTs (overall *p* = 1.000, NS) (unavailable data in the remaining cases) (Table 5).

The mitotic index was high (>5/50 high power fields or >5/5 mm^2^) in 26 (57.8%) Group 1 and 13 (81.2%) Group 2 patients; it was low in 19 (42.2%) Group 1 and in only 3 (18.8%) Group 2 cases, while data were missing in 14 Group 1 and in 5 Group 2 women. We found 22/61 (36%) GIST patients with low risk and 39/61 (64%) with intermediate–high risk (Table 5).

### 3.9. Immunohistochemistry

CD117 was expressed in 20/20 (100%) Group 2 cases and in 50/51 (98%) Group 1 GISTs (total: 70/71 cases, 99%). DOG1 was expressed in all the six (100%) tested Group 2 cases and in 12/18 (66.7%) Group 1 GISTs (total: 18 cases, 75%). CD34 was positive in 11/14 (78.6%) Group 2 and in 18/28 (64.3%) Group 1 cases (total: 29 cases, 69%).

SMA was positive in 10/22 (45.5%) GISTs, slightly more frequently in Group 2 (6/12, 50%) than Group 1 GISTs (4/10, 40%). S100 was occasionally expressed by 6/22 (27.3%) GISTs, either of Group 2 (*n* = 3/12, 25%) or 1 (*n* = 3/10, 30%). Data of Ki-67 index were available in 6/59 (10%) Group 1 cases [12,22,23,53,57,72], ranging from <1% to 40% (mean 11%), and in just one Group 2 GIST (5%) [14,52] (Table 5).

### 3.10. Survival and Recurrence

A significant difference was observed in the percentage of deceased patients between Groups 1 and 2 (*p*-value 0.017). In particular, 6/16 (37.5%) Group 2 and 5/51 (9.8%) Group 1 patients died (overall, 11/67 cases, 16%). Globally, the mean overall survival was 24.2 months, being longer for Group 2 (41 months; SD: ±52.4 months) than for Group 1 patients (18 months; SD: ±15 months).

Similarly, a significantly higher frequency of recurrent GISTs was observed in Group 2 patients (*p*-value 0.0001); in particular, 6/11 (54.5%) Group 2 and 3/44 (6.8%) Group 1 patients relapsed (overall, 9/55 cases, 16%). The mean disease-free survival was 22.1 months (SD ± 31.1 months), being longer for Group 2 (38.7 months; SD ± 57.3 months) than for Group 1 cases (17.1 months; SD ± 14.9 months). However, there was no significant statistical difference for survival between the two studied groups.

## 4. Discussion

GISTs are more frequently diagnosed in men than women, being rarely found before the age of 40 years (median age at diagnosis is in the 60s) [79]. Similar to other ovarian tumors, the most common symptoms were due to the tumor mass effect (Table 1 and Table 3).

The most common primary site of GISTs is the stomach (60–70%), followed by the small bowel (20–30%), colon–rectum (5%), and esophagus (5%) [80].

**Figure 2:**
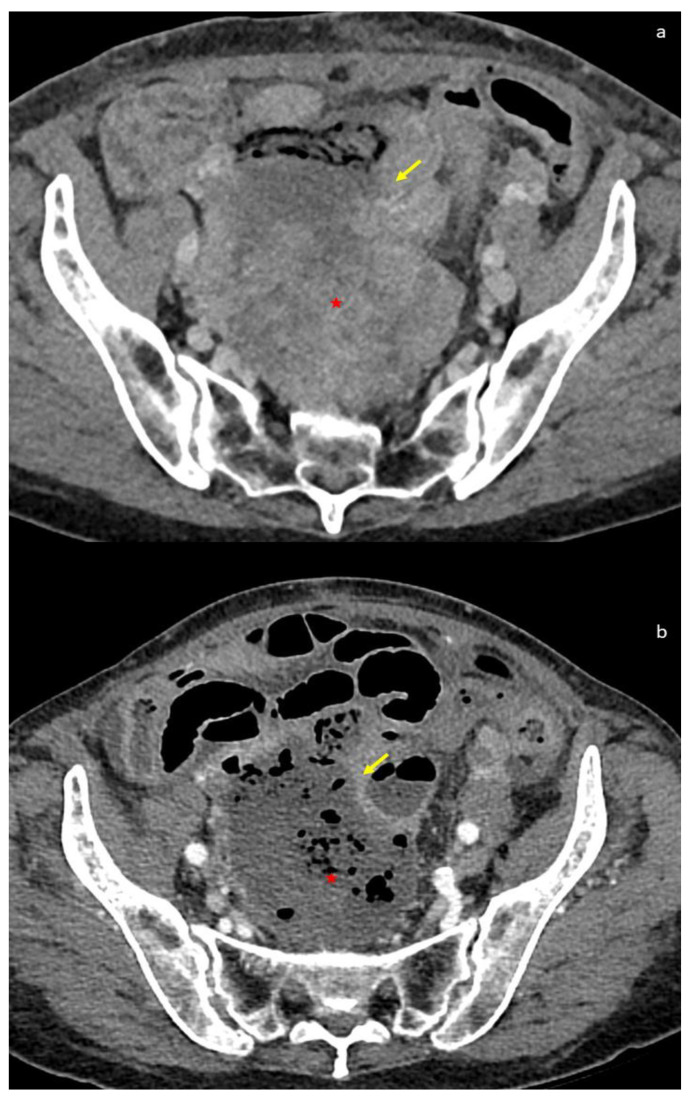
Our case: axial CT image depicting: GIST with attached ileal loop (yellow arrow) (**a**); GIST with signs of necrosis with attached ileal loop (red star) (**b**).

Differently from epithelial ovarian cancer (EOC), GISTs showed normal or slightly increased blood Ca-125 and Ca-19.9. Particularly, Group 2 patients showed increased serum Ca-125 and Ca-19.9 values more frequently than Group 1 cases (71% vs. 40% and 50% vs. 7%, respectively). In the latter cases, serological markers may cause diagnostic misinterpretation with gynecological cancers, particularly those arising from the adnexa. Not only the clinical diagnosis can be challenging in differentiating GISTs from adnexal masses but sometimes also radiologic investigation such as CT or MRI can raise problems in interpretation. CT is considered the best imaging method for diagnosing and describing tumors and for evaluating their extent and metastatic disease (Figure 2).

**Figure 3:**
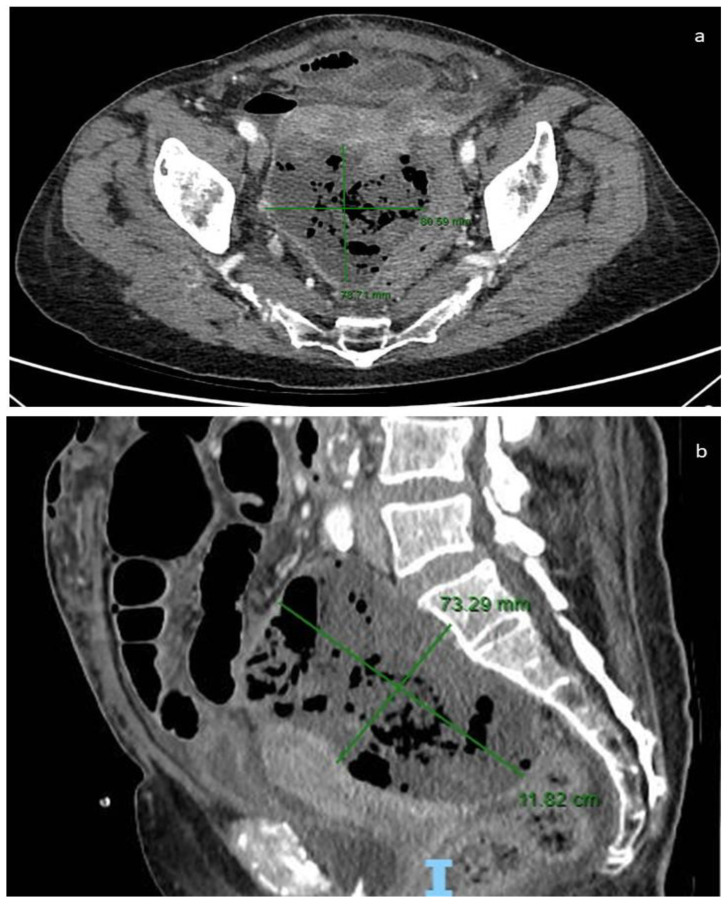
Our case: CT image with measurements: axial plane (**a**) and sagittal plane (**b**).

CT is useful for preoperatory diagnosis (CT-guided biopsy), to evaluate the full thickness of the small intestine and mesentery, and to determine the response to adjuvant therapy [81]. The density of GISTs on non-contrast CT is similar to that of the muscles and its enhancement varies. Intratumoral gas suggesting communication with the gastrointestinal lumen, calcifications, and intratumoral hemorrhage are easily identified. CT enterography may be useful for identifying small GISTs in the small bowel of patients with suspected small bowel bleeding [82]. Moreover, CT may define the size of GISTs, which correlate to stage and prognostic risk classes. GISTs larger than 10 cm were more frequently associated with peritoneal or distant metastasis, increased mortality rates, or high-grade histology. Morphologically, these GISTs frequently showed irregular growth margins with invasion to adjacent organs [83,84] (Figure 3); however, CT may fail to detect small ovarian metastases [52].

Usually, large GISTs, especially those larger than 10 cm, tend to have nonuniform density and enhancement on CT and histologically may show areas of cystic, hemorrhagic, and necrotic degeneration. On the contrary, GISTs smaller than 5 cm generally present as homogeneous lesions with regular contours.

**Figure 4:**
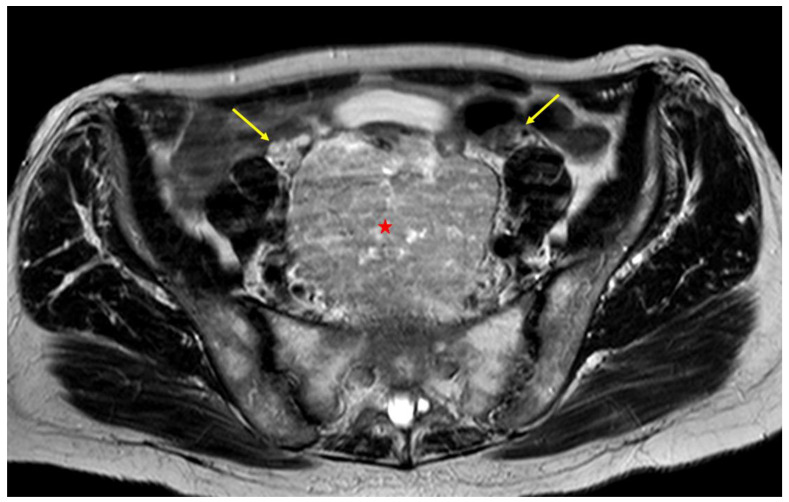
Our case: T2 axial MRI image depicting normal ovaries (yellow arrows) and GIST (red star).

According to our study, the majority of patients (85%) underwent preoperative CT. CT was associated with MRI or US in 4% and 6% of patients, respectively. MRI is commonly used to study liver metastases not detected by CT and to obtain detailed visualization of pelvic anatomy, including tumor infiltration of adjacent organs (Figure 3 and Figure 4).

**Figure 5:**
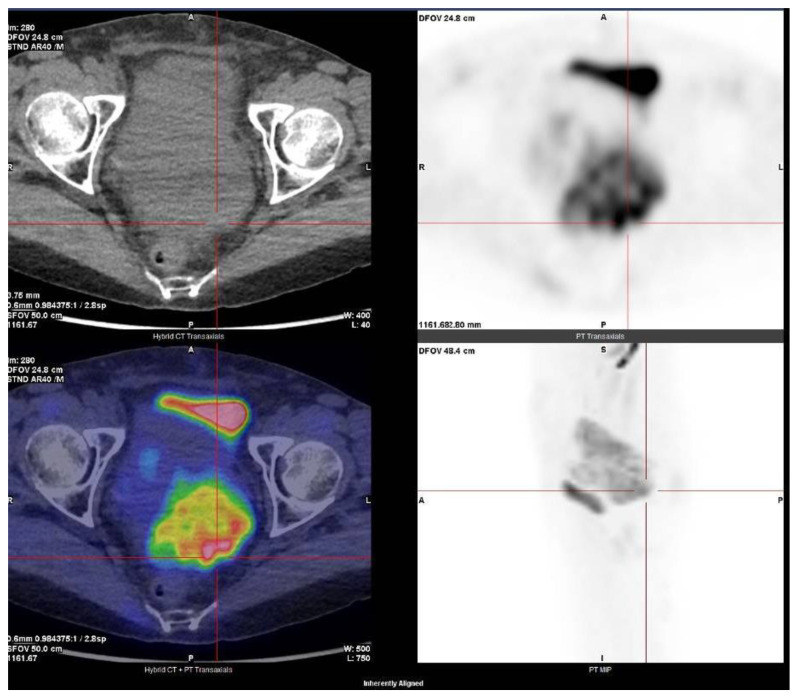
Our case: PET uptake of a GIST.

GISTs typically show low signal intensity on T1-weighted imaging (T1WI), high signal intensity on T2WI, and enhanced signal intensity on post gadolinium images [85]. Compared to CT, MRI uses quantitative parameters, such as the apparent diffusion coefficient (ADC) and the degree of enhancement, and perfusion parameters, useful for evaluating malignancy and response to treatment, respectively (Figure 4) [86]. In our review, 12% of patients underwent only MRI and in one patient MRI was associated with US. Fluorodeoxyglucose positron emission tomography (FDG-PET) is used to help in distinguishing GISTs from benign tumors, to stage the disease, and to identify areas of liver necrosis and metastases. FDG-PET is also useful for predicting and monitoring response to chemotherapy, particularly to molecularly targeted therapy (Figure 5) [81].

Abdominal US is useful for the visualization of tumors >5 cm in diameter but has a variable efficacy due to the operator skills. It has inconsistent reliability in the presence of necrosis, ulceration, and air in bowel. US is used to study hepatic metastasis and to perform image-guided biopsy. On the contrary, endoscopic US provides a more detailed study of the tumor, which could be a malignant GIST if it presents a marginal lobulation [87]. Typically, GISTs smaller than 2 cm in diameter appear as homogeneous hypoechoic lesions having a smooth margin arising from the fourth layer corresponding to the muscularis propria; however, differentiating GISTs from other submucosal tumors remain a challenge. If the GIST reaches the mucosa, superficial endoscopic biopsies may allow the correct diagnosis; endoscopic fine-needle aspiration cytology obtaining cell blocks (necessary for performing diagnostic immunohistochemical stainings) may help in cases of deeper lesions [88]. In case of Group 1 GISTs, previous traditional radiological techniques may be inadequate in the identification of the origin of a GIST located in the pelvis, whilst for ovarian masses transvaginal ultrasound (TVUS) can be more useful than CT for the proper diagnosis. On TVUS, pelvic GISTs may be solid, inhomogeneously hypoechoic, and/or lobulated. GISTs may show cystic areas due to necrosis without patient acoustic and with high vascularization at color Doppler [14]. In case of ovarian metastases from GISTs, TVUS may miss the diagnosis.

The most common ovarian metastases are due to cancers of the colon (30%), stomach (16%), appendix (13%), breast (13%), pancreas (12%), biliary tract (15%), uterine corpus (23%), or cervix (4%). Overall, these metastases represent about 16% of malignant tumors of the ovary [89]. Ovarian metastases from gastric, breast, and uterine cancers appear as solid masses at TVUS, and a typical feature is the presence of a leading central vessel; differently, those from colorectal and biliary tract carcinomas are more heterogeneous, often appearing as multilocular solid masses [90]. Commonly, ovarian metastases from GISTs are multifocal and localized to the ovarian surface, whereas metastases from other carcinomas appear deeper. However, a precise pre-surgical diagnosis of abdominal-pelvic masses still remain challenging, as tumors arising from the peritoneum, retroperitoneum, and small bowel (including GISTs) may overlap and mimic those arising from the adnexa. Indeed, all these tumors not only present with a similar symptomatology due to mass effect, but also show similar CT and MRI features, appearing as heterogeneous, septate masses, with irregular margins, vascularized at Doppler ultrasound, and partly solid and cystic, with central areas of necrosis and contrast medium enhancement [91]. In the future, the standardization of new advances in radiomics applied to CT and MRI could improve the diagnosis and the management of GISTs with significant impact on prognosis [92]. Therefore, despite multimodal diagnostics, GISTs may represent an incidental finding during surgery.

In case of a suspected adnexal tumor, exploratory laparotomy may identify the true origin of the neoplasm [12,37]. The gold standard treatment is complete surgical resection, which can be achieved in about 45–60% of cases [37,39]. In our study, surgical treatment was performed in all GIST patients, but complete tumor resection was reached in 92% of Group 1 and in 57% of Group 2 GISTs (Table 5).

GISTs are also classified according to their mitotic index, as low-, intermediate-, or high-risk tumors. We found 22/61 (36%) GIST patients with low-risk and 39/61 (64%) with intermediate–high-risk tumors. Tumor size and presence of metastasis have been demonstrated to be independent prognostic factors for OS [51]. The mean size of the tumor included in our review was 15 cm (±7.4 S.D.).

GISTs can show various histologic patterns/types: spindle (most common, about 77%), epithelioid (less than 10%), sclerosing, palisaded–vacuolated, diffuse hypercellular, sarcomatoid/dedifferentiated (de novo or after treatment with Imatinib) or mixed [42,93,94]. Results of our review confirmed the literature findings; however, histology type was not reported in 24 out of 59 (40.6%) cases of Group 1. When histology was assessed, spindle cells were present in 77% of Group 1 and in 58% of Group 2, respectively. Epithelioid cells were present in 5.3% of Group 2 and in 2.8% of Group 1. In our review, 37% of Group 2 and 20% of Group 1 GISTs were of mixed cell type (Table 5). In the literature, the mixed histotype appears to be associated with a higher stage than the pure type at presentation [95].

Epithelioid morphology is more frequently related to SDH-deficient GISTs, which are associated with higher rates of lymphovascular invasion, lymph node metastases, and syndromic cases [42]. Indeed, most GISTs are sporadic, while about 5% of cases occur in patients with genetic syndromes such as Carney–Stratakis syndrome (dyad), Carney triad syndrome (SDH-deficient GIST, pulmonary chondroma, paraganglioma), familiar Neurofibromatosis type 1 (NF-1) (multifocal, mainly small bowel SDH-deficient GISTs), and primary familial GIST syndrome [35,36].

**Figure 6:**
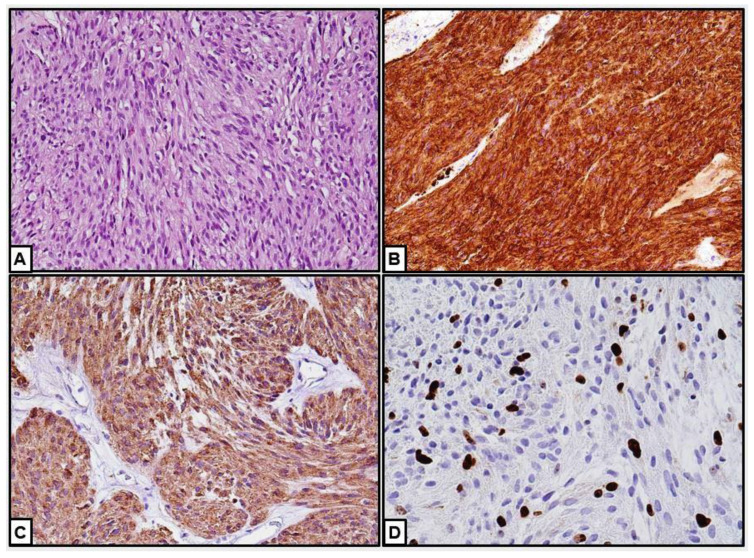
Our case: (**A**–**D**) Histological and immunohistochemical features of GISTs. (**A**) Fascicular proliferation of spindle cells with bland nuclei. Nuclear pleomorphism, necrotic areas, and mitotic figures are absent (hematoxylin and eosin, 20×). (**B**,**C**) Immunophenotype of a GIST. Tumor cells are positive for DOG-1 (**B**, 20×) and c-kit (**C**, 40×). (**D**) The proliferation index was around 10% of tumor cells (Ki-67, 40×) (previously unpublished, original photos).

Histopathological examination and immunohistochemistry are both essential for the diagnosis of GISTs. CD117 (c-kit), CD34 (less frequently expressed by epithelioid GISTs), and DOG1 result positive in a high percentage of cases and are therefore of clinical usefulness in guiding the diagnosis (Figure 6) [42,96].

Overall, approximately 88% of GISTs stain positive for both CD117 (c-kit) and DOG1; DOG-1 appears to be more sensitive and specific than CD117. In our review, CD117 was positive in all Group 2 and in 98% of Group 1 patients whereas DOG-1 was expressed in all Group 2 and in 67% of Group 1 patients (Table 5).

GISTs can be misdiagnosed as smooth muscle-tumors (such as leiomyomas or leiomyosarcoma), neural neoplasms (such as schwannomas), ovarian stromal-sex cord tumors (such as cellular fibromas or fibrosarcomas), neuroendocrine tumors, or other mesenchymal neoplasms [97,98,99,100,101,102,103,104].

In our review, CD34 was positive in 78.6% of Group 2 and in 64.3% of Group 1 patients (Table 5) and S-100 was positive in 30% of Group 1 and in 25% of Group 2 patients (Table 5).

GISTs are responsive to tyrosine kinase inhibitors like Imatinib, usually used for post-surgical treatment. In this case series 67.8% (40/59) of GIST patients received Imatinib (Table 5).

Adjuvant therapy must be carefully evaluated because even totally resected isolated GISTs can recur in approximately 40% of patients [105]. As expected, in our study, recurrence was significantly more common in Group 2 than in Group 1 patients (55% vs. 7%, respectively). Similarly, the rate of death was higher for Group 2 (38%) than Group 1 patients (10%). In contrast, Group 2 patients showed an age at diagnosis and prognosis similar to those of EOC patients [106,107].

## 5. Conclusions

From analysis of the medical literature, some conclusion can be drawn:

(1) GISTs are uncommon intestinal tumors and can metastasize to different abdomino-pelvic viscera.
(2) When metastases from GISTs occur in a female patient, an overlapping presentation with primary ovarian/adnexal tumors can make the clinical diagnosis challenging.
(3) Unlike EOC, GISTs mimicking primary ovarian tumors (Group 1) occurred in younger women, were not associated with elevated tumor markers, and had a better prognosis. In contrast, GISTs metastasizing to the ovaries (Group 2) occurred in older women than Group 1, may exhibit elevated tumor markers, and presented a worse prognosis than Group 1 patients.
(4) Usually, Group 1 GISTs originate from the small intestine. The gold standard treatment is complete surgical resection, which was achieved in 92% of Group 1 and 57% of Group 2 patients.
(5) The differential diagnoses may include many other primary and secondary ovarian tumors, so alertness to the diagnosis of GISTs is crucial; although not foolproof, CT is the preferred test for studying GISTs.
(6) A multispecialist surgical team may be necessary in the treatment of this disease, and a multidisciplinary approach and sharing of information is essential.
(7) As the final diagnosis is histological, it is fundamental to inform pathologists of the clinical presentation of the tumor (especially in case of an advanced stage disease) and of the relationship of the neoplasm with the intestines and other abdominal organs. Indeed, the lack of clinical data may not induce the pathologist to include this rare entity (especially in case of morphologically bland GISTs) in the spectrum of histopathological differential diagnoses, thus potentially favoring a misdiagnosis.
(8) Immunohistochemistry and genetic studies are crucial for the proper diagnosis, also guiding adjuvant treatment.

## Figures and Tables

**Table 1:** GISTs mimicking primary ovarian tumors (Group 1): clinical features.

Case	Age (yrs)	Site	Clinical Presentation and History	Ca-125/ Ca-19.9 (U/mL)	Treatment	Follow-Up
1. Modi et al., 2023 [8]	71	IL-J	AP; AS (mild); AM (right)	117/n (*)	Res + Om; Im	NoR; NED
2. Jawalkar et al., 2023 [53]	70s	IL	AD (12 mths), AP (acute), AM, micturition difficulty (6 mths), constipation	n/NR	Res (R0); Im	NoR; NED, 3 mths.
3. Wood et al., 2023 [12]	70	TC	AM, AP (lower), bloating/AD, normocytic An, nocturia, ovarian cystectomy (late 20s), tubal ligation incidental; AM, AS (bloody), An, anorexia	40/3 (*)	BSO + omental biopsy + Res (R0)	NoR; NED, 6 mths.
4. Kobayashi et al., 2022 [6]	79	SC	Dys, dehydratation, asthma, DVP, CI, Hyp, prerenal nephropathy, Di	646/130.2	Biopsy; TAH + BSO + Om + Res (colostomy) (R0)	Rec (pelvis, 2 mths.); DOD, 2.5 mths.
5. Huang et al., 2022 [51]	NA	NR	NR	NR	Debulking surgery (R0); Im	NED
6. Rahma et al., 2022 [20]	48	SB	Dilated stomach; stomachache, nausea	147/NR (*)	NA	NR
7. Turner et al., 2021 [56]	42	ICJ	AP (lower, 2 weeks)	15/NR	Res	NED
8. Sohail et al., 2021 [57]	60	J	Adis (lower), AM (4 mths), anorexia, WL, gout, Hyp, Dys, An	64.2/NR (*)	Debulking (R0); Im	NED
9. Shrestha et al., 2021 [16]	44	J	AP (lower, 15 days), AM, An	n/NR (*)	Res + washing (R0); Im	NoR; NED, 6 mths.
10. Hwang et al., 2020 [4]	57	J	AP (low, 3 mths), AS (bloody); An; SThy, salpingectomy (ectopic pregnancy), App, right breast mass (borderline tumor) excision	107/NR	Peritoneal biopsies, Res, retroperitoneal dissection, TAH, BSO, pelvic LND, infracolic Om, washing; Im	NoR; NED, 1 mths.
11. Oliveira Santos et al., 2020 [60]	51	ST	AD (12 mths); increased bowel movements (1 week)	118/6 (*)	NR	NR
12. Nakura et al., 2020 [47]	73	SB	Lower AP	NR	Res (R0); Im	NR
13. Deo et al., 2020 [61]	69	IL	AP (central, 4 mths), lower AM, WL	n/NR (*)	FNAC: inconclusive; LAF (3 mths); Res (R0); Im	NoR; NED, 12 mths.
14. Perrone et al., 2018: case 1 [14]	31	IL	Melena, An	<170/NR	Res (R0)	NoR; NED, 49 mths.
15. Perrone et al., 2018: case 2 [14]	77	IL	Inguinal swelling (left)	<170/NR	Res, TAH, BSO, peritoneal lumps excision (R+); Im	PD; DOD, 44 mths.
16. Perrone et al., 2018: case 3 [14]	53	IL	AP	<170/NR	Res (R0)	NoR; NED, 16 mths.
17. Perrone et al., 2018: case 4 [14]	76	IL	AP	<170/NR	Res, LSO, Om, ileal biopsies (R0)	NoR; NED, 12 mths.
18. Goyal et al., 2017 [62]	54	SB (J or IL)	AP (right, lower, 1 mths), AM	n/n (*)	FNAC: positive; Res (R0)	NR
19. Dayan et al., 2017 [63]	35	J	Infertility, excessive gas, bloating, loose stools; MIS (7 yrs before)	n/NR (*)	Res (R0)	NR
20. Baruah A., 2017 [21]	50	IL	AP, AM (lower, 4 mths), WL (5 kg/4 mths)	NR	Res	NR
21. Ijeri et al., 2016: case 1 [5]	60	IL	AM	10.9/NR	Debulking (R0)	LAF
22. Ijeri et al., 2016: case 2 [5]	55	IL	AM	161/NR	Res (R0); Im	LAF
23. Ijeri et al., 2016: case 3 [5]	50	IL	AP, AM	6.5/NR	Res (R0); Im	NoR; NED, 47 mths.
24. Ijeri et al., 2016: case 4 [5]	45	Mes	PP, AM	59.8/NR	Res, TAH, BSO (R0); Im	NoR; NED, 40 mths.
25. Ijeri et al., 2016: case 5 [5]	41	IL	AM	32.2/NR	Res (R0); Im	NoR; NED, 36 mths.
26. Ijeri et al., 2016: case 6 [5]	39	IL	PP, AM	27.4/NR	Res (R0); Im (Im/sunitinib for Rec)	Rec (multiple, abdominal, 11 mths.); DOD, 11 mths.
27. Ijeri et al., 2016: case 7 [5]	41	J	PP, AM	6.8/NR	Res (R0); Im	NoR; NED, 14 mths.
28. Ijeri et al., 2016: case 8 [5]	55	IL	AP, AM	16.4/NR	Res (R0); Im	NoR; NED, 13 mths.
29. Ijeri et al., 2016: case 9 [5]	63	SC	PP, AM	25/NR	Res (R0); Im	NoR; NED, 7 mths.
30. Ijeri et al., 2016: case 10 [5]	70	IL	AM	8.5/NR	Res (R0); Im	NoR; NED, 2 mths.
31. Akman et al., 2015: case 1 [23]	54	IL	Incidental; breast cancer	32.5/NR	Res (R0); Im	NoR; NED
32. Karaka et al., 2015 [22]	52	IL	Incidental; Hyp, Di; TAH (3 yrs before)	21/12(*)	Res, BSO, partial Om (R0); Im	NoR; NED
33. Chen et al., 2015 [30]	54	IL	AD, NF-1, An	363.3/n (*)	TAH, BSO, bilateral pelvic LND, partial Om, Res (R0); Im	NoR; NED, 42 mths.
34. Niazi et al., 2014 [65]	55	RS	AP	NR	RSO, washing, Res, LND (R0); Im	NoR; NED, 20 mths.
35. Puljiz et al., 2013 [66]	71	SC	AP, AM (3 mths), An, WL	n/n (*)	Res, TAH, BSO, Om (R0)	NoR; NED, 16 mths.
36. Lee TH, 2013 [24]	47	SC	AD, encopresis (24 mths), As increased bowel evacuations, dyspepsia, vesical tenesmus	306/n	Res, TAH, BSO; Im	NoR; NED, 12 mths.
37. Patil et al., 2012: case 4 [18]	72	HF	AM (7 mths)	n/NR	Res (R0); Im	NoR; NED, 12 mths.
38. Munoz et al., 2012: case 1 [10]	42	SB (3 J, 3 IL)	3 AP (low; 0.75–3 mths); 1 abnormal uterine bleeding (1 week), 1 incidental; AM (4 AP)	23.6/NR	Res (R0)	NoR; NED, 40 mths.
39. Munoz et al., 2012: case 2 [10]	50	SB (3 J, 3 IL)	See case 1	15/NR	Res (R0)	NoR; NED, 36 mths.
40. Munoz et al., 2012: case 3 [10]	79	SB (3 J, 3 IL)	See case 1	1.62/NR	Res (R0); Im	NoR; NED, 34 mths.
41. Munoz et al., 2012: case 4 [10]	54	SB (3 J, 3 IL)	See case 1	16.5/NR	Res (R0)	NoR; NED, 30 mths.
42. Munoz et al., 2012: case 5 [10]	46	SB (3 J, 3 IL)	See case 1	34.4/NR	Res (R+); Im	NoR; AWD, 18 mths.
43. Munoz et al., 2012: case 6 [10]	65	SB (3 J, 3 IL)	See case 1	156/NR	Res (R0)	DOC
44. Ando et al., 2011 [67]	60	J	AD, pleural effusion, AS (pseudo-Meigs syndrome), microcytic hypochromic An	517.5/2 (*)	Cystectomy, Res (R0, tumor laceration); Im	NoR; NED, 8 mths.
45. Davies et al., 2010 [69]	82	J (px)	AD, AP (right, 3 mths)	69/NR (*)	Res; Im	NED
46. Swamy et al., 2010 [70]	45	O?	AP, vomiting, AM (1 week)	n/NR	Res, TAH, BSO	NED
47. Morohashi et al., 2009 [72]	39	J	Incidental	58.5/NR	Res (R0)	NoR; NED, 0.25 mths.
48. Angioli R et al., 2009 [1]	38	IL	ADIS	38.5/NR	Res, TAH, BSO	NR
49. Matteo D. et al., 2008 [7]	56	HF	Shortness of breath, AD; TAH	55/NR	Res + left ovariectomy + App + Om (R+); Im	NoR; AWD, 6 mths.
50. McCracken et al., 2007 [73]	52	IL	AD, AM (pelvic), AP (intermittent, low), IBS, WL (mild), episodic nausea, anorexia	125/NR	Debulking (pelvic wall and sigmoid mesentery) (R0)	NoR; NED, 1 mths.
51. Pinto V. et al., 2007: case 2 [11]	76	ST	Dysuria, Adis, altered bowel habit; AM	n/n (*)	simple tumorectomy (R0)	NoR; NED, 12 mths.
52. Erkanli et al., 2006: case 2 [74]	78	SB	AP (right lower, 1 week)	n/n (*)	Res, TAH, BSO (R0)	NoR; NED, 7 mths.
53. Morimura Y. et al., 2006: case 1 [9]	69	IL	AP; TAH + left oophorectomy (20 yrs before; leiomyoma)	NR	Res	NoR; NED, 13 mths.
54. Gao et al., 2005: case 3 [76]	80	J	AM (15 days)	101.9/10.9 (*)	Res	LAF
55. Carlomagno G. et al., 2004 [2]	42	ST	AP, AM	61.8/NR	Excision (R0)	NR
56. Yeat SK. et al.,2004: case 2 [77]	48	J	AP (lower, intermittent, 4 mths), urinary frequency (4 mths), AS (minimal); TAH + colpopexy (prolapse 8 yrs before)	46.1/11.81 (*)	Res (R+); Im	NoR; AWD, 12 mths.
57. Belics Z. et al., 2003 [26]	20	TC	AM (lower), Adis	NR	Res (R0)	NoR; NED, 36 mths.
58. Powell et al., 2002 [78]	76	J (mid)	AP/P; TAH (fibroids 35 yrs before)	8/NR	Res (R0)	NoR; NED, 12 mths.
59. Powell et al., 2002 [78]	83	J (mid)	Nausea, AP (6 mths); App + LSO (childhood); uterine fibroids	NR	Res, TAH, RSO (R0)	NoR; NED, 6 mths.

Abbreviations: AD: abdominal distension; ADIS: abdominal discomfort; AM: abdominal mass; An: anemia; AP: abdominal pain; App: appendectomy; AS: ascites; AWD: alive with disease; BSO: bilateral salpingo-oophorectomy; CI: multiple cerebral/cerebellar infarcts; Di: diabetes; DOC: died of other causes (encephalopathy associated with cirrhosis soon after surgery); DOD: dead of disease; Dys: dyslipidemia; DVP: deep venous thrombosis (lower extremities); FNAC: fine needle aspiration cytology; HF: hepatic flexure of colon; Hyp: hypertension; IBS: irritable bowel syndrome; ICJ: ileo-cecal junction; IL: ileum; Im: Imatinib; J: jejunum; LAF: lost at follow-up; LND: lymph node dissection; LSO: left salpingo-oophorectomy; Mes: mesentery; MIS: melanoma in situ; mths: months; n: normal; NED: no evidence of disease; NF-1: neurofibromatosis type 1; NoR: no recurrence; O: omentum; Om: omentectomy; PD: progression of disease; PP: pelvic pain; px: proximal; R0: no residual disease after primary surgery; R+: residual disease after primary surgery; Rec: recurrence; Res: partial/segmental intestinal resection; RS: recto-sigmoid; RSO: right salpingo-oophorectomy; SB: small bowel; SC: sigmoid colon; ST: stomach; SThy: subtotal thyroidectomy (benign nodule); TAH: total abdominal histerectomy; TC: transverse colon; WL: weight loss; yrs: years. (*): normal serum CEA levels. Our case has not been included in statistical analysis.

**Table 2:** GISTs mimicking primary ovarian tumors (Group 1): pathological features.

Case	Size (cm)	M.I./Nec	WRC	AJCC Stage	Histology	c-KIT/CD34/DOG-1/SMA/S100
1. [8]	12.8	NR	3b vs. 6b	4	Spindle	+/NR/NR/NR/NR
2. [53]	23.8	L/NR	3b	3a	Spindle + Epithelioid	+/NR/+/NR/NR (°)
3. [12]	17.4	NR/Y	3b vs. 6b	3a vs. 3b	Dedifferentiated (spindle, epithelioid, pleomorphic/dedifferentiated)	−/+/+/NR/NR (°.*)
4. [6]	15	H/Y	6b	3b	Spindle (severe atypia)	+/+/−/NR/NR
5. [51]	>2	NR	NR	NR	NR	+/NR/NR/NR/NR
6. [20]	NR	NR	NR	4	Spindle	NR
7. [56]	7.5	NR	3a vs. 6a	2 vs. 3b	Epithelioid (for authors) (but spindle in photo?)	+/NR/+/NR/NR
8. [57]	18	H/Y	6b	3b	Spindle	+/−/+/−/NR
9. [16]	25	L/NR	3b	3a	Spindle	+/NR/+/NR/NR
10. [4]	15	H/NR	6b	4	Spindle	+/+/+/+/NR
11. [60]	30	NR	3b vs. 6b	2 vs. 3b	Spindle + Epithelioid	NR
12. [47]	17	NR	3b vs. 6b	3a vs. 3b	NR	+/NR/NR/NR/NR
13. [61]	8	NR	3a vs. 6a	4	Spindle	NR
14. [14]	7.5	L/NR (in 1/4 cases)	3a	2	NR	NR (*)
15. [14]	17	H/NR (in 1/4 cases)	6b	4	NR	NR (*)
16. [14]	8	L/NR (in 1/4 cases)	3a	2	NR	NR (*)
17. [14]	6.5	L/NR (in 1/4 cases)	3a	2	NR	NR (*)
18. [62]	18	H/Y	6b	4	Spindle	+/NR/NR/+/−
19. [63]	9	NR/No	3a vs. 6a	2 vs. 3b	Spindle	+/NR/+/NR/− (*)
20. [21]	15	NR	3b vs. 6b	3a vs. 3b	Spindle	+/NR/NR/NR/NR
21. [5]	24	H/NR	6b	3b	NR	+/+/−/NR/NR
22. [5]	16	H/NR	6b	3b	NR	+/−/−/NR/NR
23. [5]	3.9	H/NR	5	3b	NR	+/−/−/NR/NR
24. [5]	24	H/NR	6b	3b	NR	+/+/−/NR/NR
25. [5]	10	H/NR	6a	3b	NR	+/−/+/NR/NR
26. [5]	10	H/NR	6a	3b	NR	+/+/+/NR/NR
27. [5]	20	H/NR	6b	3b	NR	+/−/−/NR/NR
28. [5]	14	L/NR	3b	3a	NR	+/−/+/NR/NR
29. [5]	10	H/NR	6a	3b	NR	+/+/+/NR/NR
30. [5]	20	L/NR	3b	3a	NR	+/−/+/NR/NR
31. [23]	11.2	L/NR	3b	3a	Spindle + Epithelioid	+/−/NR/−/− (°)
32. [22]	7	H/Y	6a	3b	Spindle	+/NR/NR/NR/NR
33. [30]	14	H/NR	6b	3b	Spindle	+/NR/NR/NR/+
34. [65]	11	H/Y	6b	3b	Spindle	+/+/NR/−/−
35. [66]	29	L/Y	3b	4	Spindle + Epithelioid	+/−/NR/−/+
36. [24]	30	H/Y	6b	4	Spindle	+/NR/NR/NR/NR
37. [18]	25	H/Y	6b	3b	NR	+/+/NR/NR/NR
38. [10]	6	L/NR	3a	2	NR	+/NR/NR/NR/NR
39. [10]	6	L/NR	3a	2	NR	+/NR/NR/NR/NR
40. [10]	34	H/NR	6b	3b	NR	+/NR/NR/NR/NR
41. [10]	12	L/NR	3b	3a	NR	+/NR/NR/NR/NR
42. [10]	33	L/NR	3b	4	NR	+/NR/NR/NR/NR
43. [10]	10	H/NR	6a	3b	NR	+/NR/NR/NR/NR
44. [67]	20	NR/Y	3b vs. 6b	3a vs. 3b	Spindle	+/NR/NR/NR/NR
45. [69]	18	L/Y	3b	4	Spindle	+/+/NR/NR/NR
46. [70]	20	NR	3b vs. 6b	2 vs. 3b?	Spindle	+/NR/NR/NR/NR
47. [72]	8.7	L/NR	3a	2	Spindle	+/−/NR/NR/− (°)
48. [1]	17	H/Y	6b	3b	Spindle	+/+/NR/NR/NR
49. [7]	30	H/Y	6b	4	Spindle + Epithelioid; some bizarre/ pleomorphic cells	+/+/NR/−/NR
50. [73]	12	H/Y	6b	3b	Spindle	+/+/NR/NR/NR
51. [11]	17	H/Y	6b	3b	Spindle (myxoid/pseudocystic areas, degenerative changes)	+/+/NR/NR/NR
52. [74]	20	H/Y	6b	3b	Spindle	+/NR/NR/+/+
53. [9]	10	H/Y	6a	3b	Spindle + Epithelioid (focal)	+/+/NR/NR/NR
54. [76]	18	NR	3b vs. 6b	pT4	NR	+/+/NR/NR/NR
55. [2]	12	NR	3b vs. 6b	2 vs. 3b	Spindle (myxoid areas)	+/+/NR/+/−
56. [77]	20	L/Y	3b	4	Spindle	+/NR/NR/NR/NR
57. [26]	19	L/NR	3b	3a	Spindle	NR/+/NR/−/−
58. [78]	7	L/No	3a	2	Spindle	+/NR/NR/NR/NR
59. [78]	5.5	L/No	3a	2	Spindle	+/NR/NR/NR/NR

Notes: Ki-67 index: 6% (case 2), 4% (case 3), 10% (case 8), 4% (case 31), 40% (case 32), <1% (case 47). (*): Case 3: PDGFR mutation (exon 18, D842V), C-KIT wild-type; Case 14: KIT mutation (exon 11; c.1657_1668del; pY553_Q556); Case 15: KIT mutation (exon 9; c.1509_1510insGCCTA); Case 16: KIT/PDGFR wild type; Case 17: KIT mutation (exon 11; p. V559D, c.1676T>A); Case 19: KIT mutation (exon 9); Case 45: KIT mutation (exon 11). H: high (>5/50 high power fields); L: low (≤5/50 high power fields); M.I.: mitotic index; Nec: necrosis; NR: not reported; WRC: WHO risk class; Y: yes. (°): Ki-67 index: 6% (case 2), 4% (case 3), 10% (case 8), 4% (case 31), 40% (case 32), <1% (case 47).

**Table 3:** GISTs metastasizing to the ovaries (Group 2): clinical features.

Case (*)	Age (yrs)	Primary Site and OMS	Clinical Presentation and History	Treatment	Follow-Up
60. Blanco-Salazar et al. [54]	61	SB (L)	AP, AD; previous stage III breast cancer (chemotherapy + mastectomy + radiotherapy). CT: solid-cystic	TAH, BSO, Res (R0); Im	No Rec; NED, 12 mths.
61. Peralta et al., 2022 [55]	NR	NR (UMB)	NR	Mass resection (unclear type of surgery).	NR
62. Liu et al., 2022:case 4 [19]	80	SB (L)	AP, PM	TAH, BSO, Res, Om, App	Dead (unclear cause), 84 mths.
63. Liu et al., 2022:case 5 [19]	53	SB (Bi)	PM	TAH, BSO, Res (SB, sigma), Om	NR
64. Liu et al., 2022:case 6 [19]	32	SB (j) (Bi)	PM, AP	Debulking, App, Om; Im (Sunitinib for Rec)	Multiple Rec (96, 108 mths); AWD, 108 mths.
65. Saito et al., 2021 [58]	54	Rectum (L)	Constipation	Res (radical) (R0); Im	Rec: mesentery of descending colon, retroperitoneum, left ovarian artery and vein (180 mths); AWD, 180 mths.
66. Yamaguchi et al., 2021 [59]	53	Stomach (L)	Bloating, anorexia (2 mths), obesity (BMI: 41.7), anemia, asthma; Ca-125: 818 U/mL, normal CEA and Ca-19.9.	Gastric biopsy; neoadjuvant Im. (partial response after 4 mths); proximal gastrectomy + transverse colectomy + left ovariectomy (R0); Im	No Rec; NED, 10 moths.
67. Perrone et al., 2018: case 5; De leo et al., 2018 [14,52]	50	SB (ileum) (Bi)	Uterine prolapse; normal Ca-125.	Res, hysterectomy, BSO, peritonectomy (Douglas pouch and pelvic), peritoneal washing (R0); Im	No Rec; NED, 7 mths.
68. Gaballa et al., 2017 [64]	49	Intestine (°) (R)	AP, AM; Ca-125: 83 U/ml	Ovarian mass excision (R+); Im	Rec: peritoneum, adnexa, omentum, liver (4 mths; AWD, 4 mths.
69. Jindal et al., 2011 [68]	NR	Omentum (Bi)	Reduced appetite, AP (low, 3 mths), AM; hysterectomy (fibroid); normal Ca-125 and CEA.	Unclear (biopsy?) (R+); Im	No Rec; AWD, 9 mths.
70. Kinkor et al., 2008: case 2 [71]	49	SB (§)	Increasing abdominal volume, AP (persistent).	Partial tumor excision including ovarian tissue (R+)	NR
71. Beltran et al., 2006 [75]	33	SB (R)	Neurofibromatosis type 1.	Res, right ovariectomy (R0)	NR
72. Irving et al., 2005: case 1 [41]	56	SB (R)	AM	TAH, RSO, LS, Res (R0)	Dead (unclear cause), 26 mths.
73. Irving et al., 2005: case 2 [41]	44	Stomach (Bi)	AP, nausea	NR (R0)	PD; DOD, 18 mths.
74. Irving et al., 2005: case 3 [41]	54	SB (Bi)	PM; histerectomy and resection of SB “leiomyosarcoma” (possible GIST).	Unclear surgery, including bilateral ovariectomy (R+); unclear adjuvant drug treatment.	Rec: liver, omentum, abdominal scar; DOD, 78 mths (402 mths after “leiomyosarcoma”).
75. Irving et al., 2005: case 4 [41]	NA	SB (U)	NR	Resection of SB mass and ovarian mass.	Lost at follow-up.
76. Irving et al., 2005: case 5 [41]	81	SB (Bi)	NR	TAH, BSO, Res (R0); unclear adjuvant drug treatment.	Dead (unclear cause), 12 mths.
77. Gao et al., 2005: case 1 [76]	53	SB (Bi)	Fatigue, AD, weakness in lower limbs, ascites (50 days); Ca-125: 438.7 kU/L, CEA: 2.3 μg/L, Ca-19-9: 39.6 kU/L.	Res, Om (R+); Cisplatin	PD, DOD, 20 mths.
78. Gao et al., 2005: case 2 [76]	52	SB (j) (Bi)	AP, AM (lower, 20 days), moderate ascites; Ca-125: 44.6 kU/L, CEA: 4.9 μg/L.	Res, BSO	Lost at follow-up.
79. Zighelboim I. et al., 2003 [13]	31	Pelvis, NOS (R)	AP (20 days; lower, then whole pelvis) AM, urinary infection, hematuria, dysuria, ascites; Ca-125: 76.1 U/mL; CEA 0 ng/mL US: confluent echogenic pelvic masses (moderate Doppler vascularity; free-fluid). CT: many pelvic masses.	Om, left salpingo-oophorectomy, suboptimal debulking of peritoneal and mesenteric implants (R+); Im	No Rec; AWD (SD), 6 mths.

Notes: it was unclear if the metastasis was mono-or bilateral in the case of Istók et al., 2005 (case 80; only abstract retrieved) [46], so this case is not listed in the table. (°): the authors said it was a primary right ovarian GIST, but it is non-separable from intestinal loops. (§): for authors a primary ovarian GIST was not excluded, but small intestine loops were fused to other unrecognizable pelvic organs including ovary. AD: abdominal distension; AM: abdominal mass; AP: abdominal pain; App: appendectomy; AWD: alive with disease; Bi: bilateral; BMI: body mass index; BSO: bilateral salpingo-oophorectomy; CT: computed tomography scans; DOD: dead of disease; Im: Imatinib; j: Jejunum; L: left; LS: left salpingectomy; mths: months; NED; no evidence of disease; NR: not reported; NOS: not otherwise reported; Om: omentectomy; OMS: ovarian metastasis side; PM: pelvic mass; R: right; R0: no residual disease after primary surgery; R+: residual disease after primary surgery; Rec: recurrence; Res: partial/segmental intestinal resection; RSO: right salpingo-oophorectomy; SB: small bowel; PD: progression of disease; SD: stable disease; TAH: total abdominal histerectomy; U: unilateral; UMB: unclear if mono-or bilateral; yrs: years. (*): it was unclear if the metastasis was mono-or bilateral in the case of Istók et al., 2005 (case 80; only abstract retrieved) [46]; so this case is not listed in the table.

**Table 4:** GISTs metastasizing to the ovaries (Group 2): pathological features.

Case	Size (cm)	WRC	Histology	M.I./Nec	c-KIT/CD34/ DOG-1/SMA/S100
60. [54]	20	6b	Spindle	H/NR	+/NR/+/+/NR (*)
61. [55]	NR	NR	Epithelioid	H/Y	+/NR/+/NR/NR
62. [19]	14	6b	Spindle + Epithelioid	H/Y	+/+/NR/+/−
63. [19]	20	6b	Spindle + Epithelioid	H/No	+/−/NR/+/−
64. [19]	2.7	5	Spindle + Epithelioid; last recurrence: epithelioid	H/No	+/NR/+/−/NR (°)
65. [58]	12	3b	Spindle	L/NR	+/+/NR/NR/NR
66. [59]	23,4 (stomach); 13.8 (ovary)	3b vs. 6b	Spindle	NR/Y	+/+/+/−/−
67. [14,52]	7 (primary tumor); 2 and 0.5 cm (ovaries)	6a	Spindle	H/NR	+/NR/+/NR/NR (*,°)
68. [64]	14	6b	Spindle	H/Y	+/+/+/+/NR
69. [68]	6.2 (omentum)	6a	Epithelioid	H/Y	+/+/NR/−/−
70. [71]	9	3a vs. 6a	Spindle + Epithelioid	NR/NR	+/+/NR/−/− (°)
71. [75]	12	6b	Spindle	H/NR	+/+/NR/−/−
72. [41]	20	3b	Spindle; areas with signet ring cells	L/Y	+/−/NR/NR/−
73. [41]	9 (right ovary) 6 (left ovary)	6a	Spindle + Epithelioid (20%)	H/Y	+/+/NR/NR/−
74. [41]	3.8 (right ovary) 8 (left ovary)	6a	Spindle	H/Y	+/−/NR/NR/+
75. [41]	NR	1 to 3b	Spindle (small bowel); spindle + epithelioid (10%, ovary)	L/Y	+/+/NR/NR/−
76. [41]	7.4 (small bowel), 9.4 (right ovary), 8.5 (left ovary)	6a	Spindle + Epithelioid (30%)	H/Y	+/NR/NR/+/+
77. [76]	8	3a vs. 6a	Spindle	NR/NR	+/+/NR/+/+
78. [76]	19	3b vs. 6b	NR	NR/NR	+/+/NR/NR/NR
79. [13]	4	6b	Spindle	H/NR	+/NR/NR/−/NR

Notes: Ki-67 index 5%. (°) Case 5: KIT mutations (exon 11, insertion, 1732_1737 dup:p.Y578_D579dup in primary and recurrent tumor; additional KIT mutations: pV654A, exon 13 and L783V, exon 16), RB1 mutation (p.R355fs) only in last recurrence. Case 8: KIT mutation (exon 11; p.M552_W557 del; c-1653_1670del) (primary tumor and metastases) (Sanger sequencing). Case 11: KIT mutation. H: high (>5/50 high power fields); L: low (≤5/50 high power fields); M.I.: mitotic index; Nec: necrosis; NR: not reported; WRC: WHO risk class; Y: yes; (*): Ki-67 index 5%.

**Table 5:** GISTs mimicking primary ovarian tumors (Group 1) vs. metastasizing to the ovaries (Group 2): statistical analysis.

	Group 1 (*n* = 59)	Group 2 (*n* = 21)	Total (*n* = 80)	*p*-Value
**Age**				0.289
Mean (SD)	56.9 (14.8)	52.1 (13.7)	55.8 (14.6)	
NR	2	4	6	
**Site of origin**				0.174
Colon	9 (15.8%)	1 (5.6%)	10 (13.3%)	
Small bowel	45 (78.9%)	14 (77.8%)	59 (78.7%)	
Stomach–Omentum	3 (5.3%)	3 (16.7%)	6 (8.0%)	
NR	2	3	5	
**Ca-125**				0.223
High	21 (40.4%)	5 (71.4%)	26 (44.1%)	
Normal	31 (59.6%)	2 (28.6%)	33 (55.9%)	
NR	7	14	21	
**CEA**				1
Normal	18 (100.0%)	5 (100.0%)	23 (100.0%)	
NR	41	16	57	
**Ca-19.9**				0.242
High	1 (7.1%)	1 (50.0%)	2 (12.5%)	
Normal	13 (92.9%)	1 (50.0%)	14 (87.5%)	
NR	45	19	64	
**Surgery**				
Yes	57 (100.0%)	18 (100.0%)	75 (100.0%)	
NR	2	3	5	
**Residual disease after surgery**				0.007
No	43 (91.5%)	8 (57.1%)	51 (83.6%)	
Yes	4 (8.5%)	6 (42.9%)	10 (16.4%)	
NR	12	7	19	
**Chemotherapy**				0.004
No	44 (100.0%)	6 (66.7%)	50 (94.3%)	
Yes	0 (0.0%)	3 (33.3%)	3 (5.7%)	
NR	15	12	27	
**Radiotherapy**				1
No	33 (100.0%)	5 (100.0%)	38 (100.0%)	
NR	26	16	42	
**Imatinib**				0.247
No	18 (36.7%)	1 (11.1%)	19 (32.8%)	
Yes	31 (63.3%)	8 (88.9%)	39 (67.2%)	
NR	10	12	22	
**Size (cm)**				0.090
Mean (SD)	15.9 (7.6)	12.1 (6.2)	15.0 (7.4)	
NR	2	3	5	
**pT**				0.358
2	1 (1.8%)	1 (5.6%)	2 (2.7%)	
3	17 (29.8%)	7 (38.9%)	24 (32.0%)	
4	39 (68.4%)	10 (55.6%)	49 (65.3%)	
NR	2	3	5	
**GIST histotype**				0.171
Epithelioid	1 (2.9%)	2 (10.5%)	3 (5.6%)	
Spindle	27 (77.1%)	10 (52.6%)	37 (68.5%)	
Spindle–epithelioid	7 (20.0%)	7 (36.8%)	14 (25.9%)	
NR	24	2	26	
**Necrosis**				1.000
No	3 (14.3%)	2 (16.7%)	5 (15.2%)	
Yes	18 (85.7%)	10 (83.3%)	28 (84.8%)	
NR	38	9	47	
**CD117 IHC**				1.000
Negative	1 (2.0%)	0 (0.0%)	1 (1.4%)	
Positive	50 (98.0%)	20 (100.0%)	70 (98.6%)	
NR	8	1	9	
**CD34 IHC**				0.485
Negative	10 (35.7%)	3 (21.4%)	13 (31.0%)	
Positive	18 (64.3%)	11 (78.6%)	29 (69.0%)	
NR	31	7	38	
**DOG1 IHC**				0.277
Negative	6 (33.3%)	0 (0.0%)	6 (25.0%)	
Positive	12 (66.7%)	6 (100.0%)	18 (75.0%)	
NR	41	15	56	
**SMA IHC**				0.691
Negative	6 (60.0%)	6 (50.0%)	12 (54.5%)	
Positive	4 (40.0%)	6 (50.0%)	10 (45.5%)	
NR	49	9	58	
**S100 IHC**				1.000
Negative	7 (70.0%)	9 (75.0%)	16 (72.7%)	
Positive	3 (30.0%)	3 (25.0%)	6 (27.3%)	
NR	49	9	58	
**Mitotic index**				0.132
High	26 (57.8%)	13 (81.2%)	39 (63.9%)	
Low	19 (42.2%)	3 (18.8%)	22 (36.1%)	
NR	14	5	19	
**Deceased**				0.017
No	46 (90.2%)	10 (62.5%)	56 (83.6%)	
Yes	5 (9.8%)	6 (37.5%)	11 (16.4%)	
NR	8	5	13	
**Recurrence**				0.001
No	41 (93.2%)	5 (45.5%)	46 (83.6%)	
Yes	3 (6.8%)	6 (54.5%)	9 (16.4%)	
NR	15	10	25	

Abbreviations: Ca-125: carbophydratic antigen-125; CEA: carcinoembryonic antigen; CMs: centimeters; DFS: disease-free survival; DOG1: discovered on gastrointestinal stromal tumor protein 1; GIST: gastrointestinal stromal tumors; IHC: immunohistochemistry; mths: months; NR: not reported; OS: overall survival; pv: per vagina; SD: standard deviation; SMA: smooth muscle actin.
